# Special Issue “State-of-the-Art Porcine Virus Research in China”

**DOI:** 10.3390/v15020412

**Published:** 2023-02-01

**Authors:** Hongsheng Ouyang, Linzhu Ren

**Affiliations:** Key Laboratory for Zoonoses Research, Ministry of Education, College of Animal Sciences, Jilin University, 5333 Xi’an Road, Changchun 130062, China

China is one of the major countries involved in pig production and pork consumption. However, porcine viruses impose severe threats to pig farms in China. Notably, several emerging and remerging viruses have recently been first identified in China, such as African swine fever virus (ASFV), porcine deltacoronavirus (PDCoV), porcine circovirus 4 (PCV4), etc. In past decades, Chinese scientists have made significant progress in researching porcine viruses and virus-related disease control. This Special Issue contains twenty-four original studies, six reviews, and one comment, presenting “State-of-the-Art Porcine Virus Research in China”.

The original studies mainly focus on the pathogenicity, evolution, and detection of porcine reproductive and respiratory syndrome virus (PRRSV), coronaviruses, and circoviruses, which are the top porcine viruses circulating in China to date [1,2,3,4,5,6,7,8,9,10,11,12,13,14,15,16,17,18]. As reported, PRRSV regulates the expression of poly-*N*-acetyllactosamine and complex *N*-glycan in porcine pulmonary microvascular endothelial cells [1]. After infection, the glycocalyx and glycoprofiling on the cell surface are changed, leading to cell dysfunction. PRRSV-2 also upregulates the expression of miR-541-3p to promote its proliferation by negatively regulating the transcription of type I interferon (IFN I) via IFN-regulatory factor 7 (IRF7) [2]. Meanwhile, PRRSV and PCV2 coinfection and sequential infection significantly increase viral pathogenicity and cytokine responses, causing severe clinical signs, lung pathology, and death [3].

PRRSV type 2 can be divided into three phylogenetic lineages, lineage 1 (L1), L2, and L3, which can also be subdivided into several sublineages [4]. To date, L1 has one sublineage, while L2 is subdivided into L2.1 to L2.7, and L3 includes L3.1 to L3.7. Among the sublineages, sublineage 2.7 (L2.7) has the highest substitution rate, higher viral genetic diversity, and more frequent inter-lineage recombination. Further studies showed that VR2332, CH-1R, JXA1, NADC30, and NADC34-like strains play critical roles in PRRSV recombination in China, which were identified as the primary parent strains for recombinants [4,5,6,7]. Several recombination hotspots have been identified in PRRSV, among which high-frequency recombinations occur in viral glycoprotein 4 (GP4, 5.05%), followed by glycoprotein 2 (GP2, 4.89%), non-structural protein 6 (nsp6, 4.26%), nsp 7 (4.04%) proteins, etc., [4]. Notably, hotspots in the nsp 7 and the 117th–120th nucleotides in the 3′-UTR of PRRSV-2 are essential for virus replication [4,8]. These results indicate a highly recombinant rate of PRRSV in the field, mainly between vaccine strains and different co-circulated lineages.

Furthermore, codon usage contributes to genetic diversity and evolutionary dynamics [10,11,19]. For example, PCV2 can be divided into nine genotypes, PCV2a to 2i [20]. PCV2b and PCV2d are highly prevalent genotypes worldwide. In contrast, PCV2b, PCV2d, and the newly emerged PCV2e are circulating in China, indicating that PCV2 genotypes in China are more abundant [9]. Further research showed that the PCVs and PCV-like virus genomes possess an overrepresentation of AT pairs and codon usage bias caused by natural selection and mutation pressure [10,11]. In addition, host restriction factors, such as interferon-inducible transmembrane proteins (IFITMs) [21] and Mx1 [22], and cholesterol biosynthesis [23] also play vital roles in host–virus interactions. Zhou et al. reported interaction networks of PCV3 and PCV4 capsids with host proteins [12]. Yang et al. found that porcine tripartite motif protein 21 (TRIM21) can enhance PCV2 infection and host immune responses but inhibits the apoptosis of PCV2-infected cells [13]. These results further confirm that natural selection and mutation pressure caused by the host, environment, and virus itself promote epidemics and the evolution of the virus.

Moreover, this Special Issue also reports several detection methods, which are expected to detect and differentiate PRRSV, PCVs, and coronaviruses in clinical samples [14,15,16,17,18,24]. In addition, one article demonstrates that bis-benzylisoquinoline alkaloids could effectively inhibit the proliferation of porcine epidemic diarrhea virus (PEDV) and are promising for PED prevention and treatment [25]. However, more convenient, efficient, cost-effective detection methods and antiviral agents are still needed for porcine virus control.

Three reviews discuss the existing knowledge of the etiology, epidemiology, evolution, and virus–host interaction of PEDV, PCVs, and ASFV [20,26,27,28]. Another two reviews mainly focus on strategies for preventing and controlling porcine viruses, especially ASFV, classical swine fever virus (CSFV), and porcine enteric coronaviruses [29,30]. These reviews provide valuable insights for preventing and controlling the diseases caused by porcine viruses.

We acknowledge all authors for their contributions to this Special Issue. The research results and reviews summarized in this Special Issue may provide updated views on porcine viruses and reflect the significant contributions of Chinese scholars to the field. However, due to the evolution of porcine viruses in the host and susceptible animals, we need to continue monitoring the epidemiology and evolution and develop efficient and cross-protective multivalent vaccines. Furthermore, host restriction factors and antiviral agents identified by high-throughput screening, such as proteomics, small-molecule drugs, and CRISPR/Cas 9 libraries, are also crucial directions for future research.
